# The effect of transcranial direct current stimulation (tDCS) on locomotion and balance in patients with chronic stroke: study protocol for a randomised controlled trial

**DOI:** 10.1186/s13063-017-2219-6

**Published:** 2017-10-23

**Authors:** M. Geiger, A. Supiot, R. Zory, P. Aegerter, D. Pradon, N. Roche

**Affiliations:** 1 Inserm Unit 1179, Team 3: Technologies and Innovative Therapies Applied to Neuromuscular diseases, UVSQ, CIC 805, Physiology-Functional Testing Ward, AP-HP, Raymond Poincaré Teaching Hospital, Garches, France; 2 Laboratory of Human Motricity, Sport, Education and Health (EA 6312), University of Nice Sophia Antipolis, Nice, France; 3 Assistance Publique-Hôpitaux de Paris, Hôpital Ambroise Paré, Unité de Recherche Clinique (URC), Boulogne, France; 4 CIAMS, Université Paris-Sud, Université Paris-Saclay, 91405 Orsay Cedex, France; 5 CIAMS, Université d’Orléans, 45067 Orléans, France

**Keywords:** Stroke, Transcranial direct current stimulation, Gait, Motion analysis, Balance

## Abstract

**Background:**

Following stroke, patients are often left with hemiparesis that reduces balance and gait capacity. A recent, non-invasive technique, transcranial direct current stimulation, can be used to modify cortical excitability when used in an anodal configuration. It also increases the excitability of spinal neuronal circuits involved in movement in healthy subjects. Many studies in patients with stroke have shown that this technique can improve motor, sensory and cognitive function. For example, anodal tDCS has been shown to improve motor performance of the lower limbs in patients with stroke, such as voluntary quadriceps strength, toe-pinch force and reaction time. Nevertheless, studies of motor function have been limited to simple tasks. Surprisingly, the effects of tDCS on the locomotion and balance of patients with chronic stroke have never been evaluated. In this study, we hypothesise that anodal tDCS will improve balance and gait parameters in patients with chronic stroke-related hemiparesis through its effects at cortical and spinal level.

**Methods/design:**

This is a prospective, randomised, placebo-controlled, double-blinded, single-centre, cross-over study over 36 months. Forty patients with chronic stroke will be included. Each patient will participate in three visits: an inclusion visit, and two visits during which they will all undergo either one 30-min session of transcranial direct current stimulation or one 30-min session of placebo stimulation in a randomised order. Evaluations will be carried out before, during and twice after stimulation. The primary outcome is the variability of the displacement of the centre of mass during gait and a static-balance task. Secondary outcomes include clinical and functional measures before and after stimulation. A three-dimensional gait analysis, and evaluation of static balance on a force platform will be also conducted before, during and after stimulation.

**Discussion:**

These results should constitute a useful database to determine the aspects of complex motor function that are the most improved by transcranial direct current stimulation in patients with hemiparesis. It is the first essential step towards validating this technique as a treatment, coupled with task-oriented training.

**Trial registration:**

ClinicalTrials.gov, ID: NCT02134158. First received on 18 December 2013; last updated on 14 September 2016. Other study ID numbers: P120135 / AOM12126, 2013-A00952-43.

**Electronic supplementary material:**

The online version of this article (doi:10.1186/s13063-017-2219-6) contains supplementary material, which is available to authorized users.

## Background

Stroke is the primary cause of morbidity and the third cause of mortality (50,000 deaths per year) in industrialised countries. Stroke causes multiple impairments, including motor deficits, with a loss of voluntary movement, abnormal movements and changes in muscle tone that reduce balance and alter gait [1, 2]. Postural symmetry is altered, as indicated by changes in the position and stability of the centre of pressure (COP). Studies using force platforms have demonstrated an increase in postural oscillations, as well as a shift of the COP towards the non-hemiparetic limb (asymmetry of weight distribution) [3–7]. Eng and Chu (2002) showed that 79 to 87% of patients with hemiparesis support less weight through their hemiparetic limb (25 to 43% of body weight) [8]. Nardone et al. (2009) found a relationship between the degree of postural asymmetry measured by stabilometry and gait capacity [9]. Moreover, it has been shown that postural control and static equilibrium following stroke directly influence spatiotemporal gait parameters. For example, gait velocity in patients with hemiparesis has been shown to relate to the quality of static equilibrium, weight transfer capacity towards the hemiparetic limb and weight distribution between the limbs during gait [1]. There is also a relationship between gait efficacy measured by the Motor Assessment Scale and the degree of weight transfer on the hemiparetic limb [10]. Interestingly, patients with hemiparesis limit the velocity and amplitude of their movements, inducing internal perturbations of posture [5].

As well as impairing balance, stroke alters gait. Patients with stroke typically have: (1) altered kinematics including reduced hip flexion, knee flexion, hip extension, increased knee extension during stance and reduced ankle dorsiflexion during swing [11–13] and (2) spatiotemporal changes such as asymmetry in the duration of support and swing phases between the two legs [10, 12, 14, 15], reduced gait speed, stride length and cadence [1, 11, 16]. These disorders are mainly due to paresis and spasticity, especially spasticity of the rectus femoris and triceps surae muscles [12, 17]. Currently, three-dimensional (3D) gait analysis is the ‘gold standard’ method to quantify gait parameters and to guide treatment [16, 18–22]. This technique consists of simultaneously quantifying spatiotemporal, kinematic and kinetic gait parameters. In addition, the displacement of the centre of mass (COM) during gait can also be evaluated [23], providing useful information regarding the control of gait [23–25]. Studies of the displacement of the COM in patients with hemiparesis [23, 24] have shown that its displacement is altered compared with healthy subjects, who have stereotypical trajectories.

The primary motor cortex (M1) and the cortico-spinal tract play a greater role in the control of locomotion in humans than in other mammals. Many studies suggest that gait is accompanied by an increase in cortical activity [26, 27]. Several authors have demonstrated that during gait, cortico-spinal neurone activity occurs in parallel with, or perhaps partially controls, the activity of spinal motoneurones [28–30]. In patients with hemiparesis, Dobkin et al. (2004) showed a relationship between improvements in gait and activation capacity of the different cortical areas representing the lower-limb muscle groups involved in gait [31]. Therefore, any method that facilitates activation of the cortical neurones at the origin of the descending tracts that control the spinal motoneurones of the hemiparetic limb (final common pathway) could improve motor performance in patients with cortical or subcortical lesions causing gait and balance impairments.

Transcranial direct current stimulation (tDCS) was developed in humans by Priori in 1998 and by Nitsche and Paulus in 2000 [32, 33]. The latter authors showed that, when applied over the motor cortex, tDCS modifies cortical excitability depending on the polarity used. When the anode is placed over the motor cortical area to be stimulated, and the cathode above the contralateral eye, tDCS (thus termed anodal) increases cortical excitability, both during and following the stimulation, demonstrated by an increase in the amplitude of the motor-evoked potential (MEP) generated by TMS. Conversely, when the cathode is placed over the motor cortical area to be stimulated, and the anode above the contralateral eye, cortical excitability is reduced. Moreover, the effects of tDCS persist after the stimulation (named post effects) [33]. The duration of the post effects depends on the duration and intensity of the stimulation [32, 33]. Furthermore, tDCS is safe, easy to use, well tolerated, and has minor side effects such as (1) a sensation of itching under the stimulating electrode, (2) post-stimulation headache and (3) mild nausea, which is rare [34].

The effects of tDCS are not limited to the motor cortex below the stimulating electrode. During stimulation, anodal tDCS also induces changes in the excitability of spinal neuronal circuits [35–37]. Roche et al. (2011, 2012) showed that 20 min of anodal tDCS increased homonymous recurrent inhibition on the α motoneurones of the soleus muscle during the stimulation [36], and decreased lumbar propriospinal facilitation [37] during and after cessation of the cortical stimulation in healthy subjects. However, cathodal tDCS did not modify the excitability of spinal neuronal circuits [36, 37]. Jeffery et al. (2007) also found that anodal tDCS over the area of the tibialis anterior increased cortical excitability in healthy subjects (evaluated using TMS) [38] while cathodal tDCS did not. Anodal tDCS over the M1 leg area (1 mA for 20 min) has been shown to improve dynamic balance compared with a placebo condition [39], as well as reducing reaction time and increasing toe-pinch strength in healthy subjects [40]. Tanaka et al. (2009) thus concluded that anodal tDCS would be a useful tool for the neurorehabilitation of patients with motor disorders of the lower limbs [40].

The results of these different studies strongly suggest that anodal tDCS is a more appropriate stimulation configuration than cathodal tDCS to improve complex motor tasks, such as gait and balance in stroke patients, since it increases cortical excitability and modifies spinal circuit excitability, improving balance [41] and preventing abnormal muscle activation during gait [42]. Indeed, Katz and Pierrot-Deseilligny (1982) showed that during low intensity contractions of the soleus muscle, homonymous recurrent inhibition of the motoneurones did not increase as much as in healthy subjects [41]. Since anodal tDCS increases homonymous recurrent inhibition on the α motoneurones of the soleus muscle [36], it might improve static balance. Marque et al. (2001) showed that the excitability of the lumbar propriospinal system is abnormally increased on the hemiparetic side [42]. They suggest this increased excitability may be the cause of inappropriate activity of the quadriceps muscle during gait, thus theoretically, tDCS should improve gait in patients with hemiparesis by decreasing abnormal muscle activities.

This hypothesis is in accordance with previous results in stroke patients. Chang et al. (2015) found improvements in the lower-limb subscale of the Fugl-Meyer Assessment and the lower-limb Motricity Index after 10 sessions of anodal tDCS (2 mA for 10 min) over 2 weeks, but not in the Functional Ambulatory Category, Berg Balance Scale or spatiotemporal gait parameters [43]. Tanaka et al. (2011) found that anodal tDCS (2 mA for 10 min with the anode placed above the hemiparetic lower-limb M1 representation) significantly increased quadriceps strength in the paretic limb during the stimulation, compared with a placebo condition [44].

In patients with chronic stroke, only two case studies have recently been reported. Dumont et al. (2015) showed that a single session of tDCS (2 mA for 20 min) combined with treadmill training improved static balance in a patient 4 years after the stroke [45]. This case study is interesting; however, since there was no placebo condition, it is difficult to determine the respective contributions of the anodal tDCS and the treadmill training on the improvement in static balance. Nevertheless, this result constitutes an argument for further study of the effects of anodal tDCS on balance in patients with chronic stroke. The second case study of a patient 1 year post stroke showed that repeated sessions (five consecutive days for 3 weeks) of anodal tDCS (2 mA for 20 min) combined with functional electrical stimulation improved performance of the 10-m Walking Test and the Timed Up and Go Test (TUG) [46].

The results of these studies suggest that tDCS may be a useful treatment for patients with subacute and chronic stroke; however, the methodologies are highly variable. In some studies, tDCS is combined with an interventional technique such as treadmill training, rehabilitation or functional electric stimulation; sometimes it is administered for a single session, and sometimes repeated sessions. Moreover, although the case studies are interesting, they do not constitute proof of the effectiveness of anodal tDCS on locomotion and static balance in patients with chronic stroke. Therefore, we plan to accurately assess the effects of a single session of anodal tDCS versus placebo on locomotion and static balance in chronic stroke patients, in order to provide robust information regarding the possible impact of anodal tDCS on locomotion following stroke.

As mentioned above, since the activity of the corticospinal neurones from the M1 motor cortex that control movement of the lower limb increases during gait, we hypothesise that anodal tDCS will improve gait and balance parameters in patients with hemiparesis by increasing cortical excitability both during and after stimulation, and modifying the excitability of spinal circuits mainly during stimulation.

The main aim of this randomised controlled trial is, therefore, to show that a single session of anodal tDCS can decrease the variability of the displacement of the COM during gait, during a static-balance task and during a gait-initiation and obstacle-crossing task in patients with chronic stroke. According to Bikson et al. (2013), tDCS specifically enhances the task performed during the stimulation [47]. Therefore, we will assess the primary outcomes before, during and after the tDCS.

The secondary objectives are:

- Firstly, to show that compared to placebo condition, anodal tDCS improves spatiotemporal, kinematic and kinetic gait parameters, as well as the ability to follow a trajectory or cross an obstacle
- Secondly, to show that, compared to a placebo condition, anodal tDCS improves performance on functional tests and clinical tests (assessment of strength and spasticity) that will be not performed during the stimulation. This will determine whether anodal tDCS only improves tasks executed during the stimulation, or if the effects can transfer to other tasks (for example, the paretic upper limb)
- Thirdly, to show if changes induced by anodal tDCS are correlated with the patient’s profile (based on clinical evaluation scales, performance on functional tests and the time since lesion onset)

## Method/design

### Study design

This is a prospective, randomised, placebo-controlled, cross-over, double-blind, single-centre study over a period of 36 months.

Each patient included will participate in three visits.

Visit 1 (V1): inclusion and baseline assessment visit. Patients who fulfil all the inclusion and non-inclusion criteria and who have signed informed consent will be included in the study. During this visit, all the clinical and functional evaluations, the instrumented gait analysis and instrumented balance task will be carried out. The patient will also complete all the clinical scales and self-evaluation questionnaires. Furthermore, blood samples will be taken in order to carry out genetic studies and identify carriers of the Val66Met polymorphism in the brain-derived neurotrophic factor gene, since Val66Met reduces the effects of all types of non-invasive brain stimulation [48, 49].

**Fig. 1:**
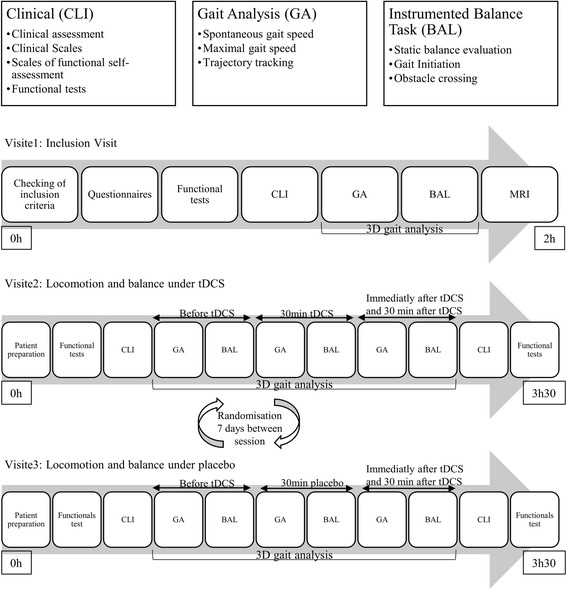
The study schedule. Study timeline from the inclusion visit (V1) to the end of the study (V2 and V3). Patients will undergo clinical evaluations (CLI), gait analysis (GA) and an instrumented balance evaluation (BAL)

Visits 2 and 3 locomotion/balance (V2 and V3): anodal tDCS or placebo visits. During these visits, before the cortical stimulation (anodal or placebo), all the functional and clinical data will be collected. A 3D gait analysis and instrumented balance task will be carried out before and during the 30 min of stimulation, as well as immediately after the end of the stimulation and 30 min later. Functional and clinical data will be collected again following the stimulation. The order of the placebo and anodal tDCS visits will be randomised. The visits will be 7 days apart. The study schedule is shown in Fig. 1 and the evaluations conducted during the three visits are shown in Table 1.

**Table 1:** Tests and evaluations during each visit

Test	V1	V2	V3
Inclusion/exclusion criteria	X		
Informed consent	X		
Randomisation	X		
Stimulation (anodal or placebo)		X	X
Evaluation of passive range of motion (manual goniometer)	X	X	X
Evaluation of spasticity (modified Ashworth Scale)	X	X	X
Evaluation of strength (Medical Research Council)	X	X	X
Gait quality: ABILOCO scale	X		
Berg Balance Scale	X		
FIM	X		
SF36	X		
Time to ascend and descend a flight of ten 11-cm-high stairs	X	X	X
Timed Up and Go Test	X	X	X
Six-minute Walk Test	X	X	X
Ten-metre Timed Walk – maximal	X	X	X
Box and Block test	X	X	X
3D gait analysis	X	X	X
3D trajectory tracking	X	X	X
Instrumented gait and balance tests	X	X	X

Abbreviations: The Short Form Health Survey (SF36) Functional Independence Measure (FIM)

### Stroke diagnosis

Participants will be individuals with chronic stroke (more than 6 months). For the purpose of this study, stroke is defined, according to the World Health Organisation as ‘a rapid onset event of vascular origin reflecting a focal disturbance of cerebral function, excluding isolated impairments of higher function and persisting longer than 24 h’ [50].

### Inclusion criteria

Patients will be included if they fulfil the following inclusion criteria: adult man or woman aged 18 years or older, with hemiparesis following unilateral hemispheric cerebral lesions of vascular origin more than 6 months previously, able to walk for 10 min non-stop with no gait aids, having provided informed consent.

### Exclusion criteria

Patients will be excluded if they have any of the following: a pacemaker, claustrophobia (for the magnetic resonance imaging (MRI)), aphasia or cognitive difficulties that could interfere with comprehension of instructions, neuro-orthopaedic surgery to the lower limb less than 6 months previously, concomitant progressive disease, epileptic fit less than 1 year prior to the date of inclusion, intracerebral metal clip, non-affiliation to the social security regime, being under guardianship.

### Participant recruitment

Patients will be recruited from inpatient wards and outpatient consultations in the physical medicine and rehabilitation department of our university hospital. Once potential patients have been identified, the investigator or his assistant will approach them to provide information about the study, both in written and verbal form, only after the patient has indicated interest in participating.

### Randomisation method

Randomisation will be carried out at the end of the inclusion visit. A randomisation list will be uploaded on a dedicated server (CleanWeb) using ‘Randoweb’ software and made available on a website for centralised randomisation.

### Blinding

In order to ensure patient blinding, one electrode will be placed over the motor cortex of the affected hemisphere and one additional electrode on the contralateral orbit in accordance with the placement procedures described below. When the current is initiated, patients traditionally report a sensation of pruritus under the active electrode for 2 min. In order to mimic this sensation, in the placebo condition, the patient will receive 120 s of current (which is less than the 3 min necessary to induce effects [33]).

In order to maintain the blinding of the investigator and assistant, an independent physician will set up the tDCS in either anodal or placebo mode, this physician will not be involved in recording, collecting or processing the data.

### Intervention

Anodal tDCS: the anode will be placed over Cz of the international electroencephalogram (EEG) 10–20 system and the cathode above the contralateral eye [51]. Rectangular electrodes (25 cm^2^) inserted in a saline-soaked sponge will be used. The stimulation intensity will be set to 2 mA for 30 min with a current density of 0.07 C/cm^2^. These stimulation criteria are well below the threshold for tissue damage [52]. The target intensity will be reached in 8 s and, at the end of the 30 min, it will be reduced over 8 s.

Placebo tDCS: the electrodes will be positioned exactly as for anodal tDCS; however, the current will only be delivered for 150 s, 120 s at the beginning and 30 s at the end of the 30 min in order to mimic the possible sensation of pruritus perceived during the increase and decrease in current intensity. Nitsche and Paulus (2000) previously showed that 3 min of tDCS were necessary to induce a post effect [33].

There will be an interval of 7 days between the two stimulation conditions.

### Characterisation of patients (inclusion visit only)

Several evaluations will only be carried out at the inclusion visit, in order to characterise the patients: the ABILOCO scale composed of 13 items to evaluate gait quality; the Berg Balance Scale, an objective measure of balance and risk of falls consisting of 14 items; the Functional Independence Measure (FIM), a measure of independence in activities of daily living; and The Short Form (36) Health Survey (SF36) questionnaire of health status that is often used as a measure of quality of life.

### Primary outcomes

#### Before, during, immediately after, and 30 min after tDCS cessation

- Variability of the COM

As previously indicated, the variability of COM displacement is a strong marker of dynamic stability during locomotion and it is altered in patients with stroke.

The primary outcome measure of this study is thus the assessment of the variability of the displacement of the COM during gait and during trajectory tracking over the ground, evaluated by 3D gait analysis and the variability of the displacement of the COP during a static-balance task on a force platform. It will be recorded once during the inclusion visit then four times during the stimulation visits: before, during, immediately after and 30 min after cessation of stimulation. A reduction in the variability of COM displacement should result in an improvement of spatiotemporal, kinematic and kinetic gait parameters.

The 3D gait analysis will be conducted with a 3D optoelectronic system (Motion Analysis Corporation, Santa Rosa, CA, USA, sampling frequency 100 Hz) with eight optoelectronic cameras. Thirty markers will be placed on the patient’s body (following the Helen Hayes model commonly used by the biomechanical community for gait analysis [53]). The relative displacement of each segment will be calculated from these coordinates (flexion/extension, abduction/adduction, internal/external rotation). The marker trajectories will then be filtered using a fourth-order zero-lag Butterworth low-pass-filter, with a 6-Hz cut-off frequency [54]. The data from two force platforms combined with the kinematic and anthropometric data will be used to evaluate the displacement of the COP and COM during gait and balance tasks.

- Instrumented gait task

Ten trials of gait at a comfortable speed and 10 trials at maximal speed will be recorded

- Instrumented balance task

Three balance conditions of differing complexity will be evaluated to assess the effects of tDCS on postural capacity:

1. Static (i.e. orthostasis): in standing, with both feet on a force platform separated by a standardised distance, patients will be asked to remain as immobile as possible for 60 s. Two sensory conditions will be tested: with (eyes open: EO) and without (eyes closed: EC) visual information
2. Analysis of the trajectory of the centre of foot pressure coupled with the kinematic analysis of the body segments recorded by an optoelectronic movement analysis system will allow us to determine the characteristics of the stability of bipedal posture in these different sensory conditions
3. Static-dynamic transition (i.e. initiation of the first step): while standing, with both feet on a force platform, patients will be asked to initiate 10 steps with the paretic foot and 10 steps with the non-paretic foot. Analysis of the trajectory of the centre of foot pressure coupled with the kinematic analysis of the body segments recorded by an optoelectronic movement analysis system and electromyography (EMG) analysis of the muscles involved will allow us to determine the characteristics of postural stability during the transition from a static to a dynamic posture

### Secondary outcomes

#### Before, during, immediately after and 30 min after tDCS cessation

The same 3D gait analysis described above will be used to evaluate the effect of anodal tDCS on kinematic and kinetic gait parameters, trajectory tracking and obstacle crossing.

- Gait analysis:

Ten trials of gait at a comfortable speed and 10 trials at maximal speed will be recorded in order to evaluate the spatiotemporal, kinematic, kinetic and EMG parameters during gait. Surface EMG will also be performed during the gait analysis. These data will be used to define the patterns of activation of the principle superficial muscles (rectus femoris, vastus lateralis, medial hamstring, biceps femoris, tibialis anterior, medial gastrocnemius and soleus) activated during gait. The EMG data will be used to quantify the activity of the agonist and antagonist muscles (of the hip, the knee and the ankle) in order to determine a possible interaction between muscle activity, variability of COM displacement and joint kinematics

- Instrumented path-following task (trajectory tracking)

From a standing start, the patient will be asked to follow a 2.5-cm-wide strip on the ground, walking with one foot on each side of the strip. The 8-m-long strip will be positioned so as to form a figure of 8. The distance of the trajectory of the COM during this task, as well as its oscillations with respect to the strip, will be quantified. The trajectory length and COM oscillations before, during, immediately after and 30 min after cessation of stimulation will be compared to determine whether the patients’ dynamic stability during the task has been modified by the tDCS

- Instrumented evaluation of obstacle crossing

From a standing start with one foot on a force platform, the patient will be asked to step over an 11-cm-high, 2-m-wide bar leading with the paretic limb (three trials) and the non-paretic limb (three trials). The time to cross the obstacle, the number of contacts with the obstacle and the height of the foot relative to the obstacle will be determined, as well as the trajectory of the centre of pressure (COP) until the foot is lifted. The patient will be asked to stand still after crossing the obstacle

- Before and after the tDCS: clinical evaluation

Passive range of motion (manual goniometer) of the hip, knee and ankle joints of the hemiparetic lower limb, spasticity (modified Ashworth Scale) and muscle strength (Medical Research Council) of the hip, knee and ankle flexor and extensor muscles of the hemiparetic lower limb will be evaluated before and after stimulation. Functional gait and balance scales and functional tests will also be evaluated (described below) before and after stimulation

- Functional clinical tests

Four functional clinical tests of gait and balance will be evaluated: the time to ascend and descend a flight of ten 11-cm-high stairs; the Timed Up and Go Test that evaluates postural transitions, gait, balance, turn-around and risk of falls; the Ten-metre Timed Walk that evaluates gait speed will be carried out at maximal speed; and the 6-minute Walk Test that evaluates the distance walked in 6 min, providing an indication of endurance capacity

- Tests to verify if the upper limb is affected by anodal tDCS

The Box and Block test evaluates unilateral gross manual dexterity. It will be carried out in order to verify if the effects of the stimulation are limited to the motor tasks carried out during the stimulation or also occur in similar tasks such as functional tests (mentioned above) or tasks carried out by another limb.

#### Relationship between effect of tDCS and patient’s level of function

All the data collected during the clinical and functional tests and the gait analysis will be used to establish the functional level of each patient and to check whether the effects of tDCS are the same for all patients with different functional levels recovery or if they depend on initial functional level.

### Statistical analysis

The details of calculation of the COM and the COP are available in the ‘Appendix’ section.

The sample size was calculated from the data by Bonnyaud et al. (2016) [24]. Bonnyaud et al. [24] studied the raw trajectory length of patients with hemiparesis compared to a group of healthy subjects during a gait task (Timed Up and Go Test), which is very similar to what we will do by evaluating the variability of the COM during gait. Based on the data of Bonnyaud et al. [24], there was a difference of 99.5 cm in the trajectory lengths of the patients with stroke and the healthy subjects. In the present study, we hope to find a reduction in this difference following anodal tDCS; however, following placebo tDCS, there should be no difference. To determine the size of the sample of patients, we used the standard deviations of the patients with hemiparesis included in the study by Bonnyaud et al. [24].

In order to obtain a 50 to 60% reduction of the difference in COM variability between anodal and placebo tDCS with a power of 90% and a moderate to large effect size, a sample of 39 subjects is necessary. We therefore decided to include 40 patients in order to cover any drop outs. All the calculations were carried out using XLSTAT 2016® with a threshold of 90% power and a significance of *α* = 0.05.

#### Analysis of outcome measures

Patient characteristics (age, gender, etc.) will be described by means, medians and standard deviations for continuous numeric parameters and by frequency tables with 95% confidence intervals for qualitative parameters.

A chi-squared test with Yates’ correction or the Fisher’s exact chi-squared test will be used to compare the distributions of qualitative variables.

There will be two possibilities for quantitative and ordinal variables:

1. If the data are close to a normal distribution, means will be compared using Student’s *t* test

Comparison of the means or medians will be carried out for all the functional clinical data and the data determined following the movement analyses during the different evaluations. Repeated measures analysis of variance (ANOVA) and/or analysis of covariance as well as *t* tests (and/or non-parametric sign tests), corrected using the Scheffé adjustment, will be used for multiple comparisons.

2. If the variables do not follow a normal distribution or if the small number of subjects studied does not allow the normality to be determined, we will use non-parametric tests such as Wilcoxon for the comparison of two variables, or Kruskall-Wallis for the comparison of more than two variables

All tests will be carried out with a bilateral threshold of first order of 5%.

### Data monitoring

This research carries a level-B risk.

The clinical research associate (CRA), who represents the sponsor, will visit the investigating centre at a frequency that corresponds to the patient follow-up specified in the protocol. Several monitoring visits will be carried out:

- An opening visit in each centre: before the first inclusion, to set up the protocol and make acquaintance with the various participants involved
- During the protocol: the Case Report Forms will be reviewed by the CRA. The principal investigator, who includes and follows the persons involved in the research, will accept regular visits from the CRA.
  - o During these visits, and in accordance with the Good Clinical Practice, the following elements will be reviewed:
  - ■ Compliance with the protocol and defined procedures for research
  - ■ Audit of informed consent by the patients
  - ■ Review of source documents and comparison with data reported in the Case Report Forms: accuracy of data, missing data, consistency of data according to the rules laid down by DRCD procedures
- Closing visit: review and archiving of the biomedical research documents

### Adverse event monitoring and reporting

Adverse events will be carefully monitored during this study. The team at the clinical intervention sites will monitor and report all minor and serious adverse events that occur, if any, from enrolment to the end of the study. The following adverse events could occur will be (effects of tDCS and risk of fall during gait):

During the stimulation: certain effects may be perceived such as : (1) a sensation of pruritus under the active electrode during the first 2 min of switching on the current, (2) in rare cases, headache during stimulation, (3) prickling sensations and (4) a sensation of fatigue.

After stimulation: headaches, nausea and nocturnal insomnia following the stimulation.

## Discussion

### Impact

Comparison of the effects of anodal and placebo tDCS on the different motor functions should demonstrate that anodal tDCS significantly improves gait and balance in chronic stroke by reducing the variability of the COM during gait, static and static-dynamic balance tasks. This study should demonstrate that anodal tDCS improves kinematic and kinetic gait parameters, and performance on functional tests, and reduces the clinical symptoms of stroke (reduced spasticity and increased strength). We will also determine: (1) if the changes induced by anodal tDCS are correlated with the patient’s level of function according to the clinical evaluation scales and (2) if there is a relationship between functional performance and improvements following anodal tDCS and the time since lesion. Moreover, comparison of the effects of tDCS over time will allow the kinetics of action of the stimulation to be determined. Furthermore, since each task is evaluated by several different methods, the comparison of the effects of anodal tDCS on the task carried out during the stimulation with a similar task not tested during the stimulation will determine if the effects of tDCS on a motor task are potentialised by the practice of a task during stimulation as suggested by Bikson et al. (2013) [47]. However, if the results show that tDCS has no effect on the variables analysed, the comprehensive data collection will allow analysis of whether the lack of effects depends on the profiles or the level of performance of the patients. If no robust explanation is found, this would suggest that anodal tDCS has no effect on gait and balance. In this case, further studies should investigate other stimulation conditions (bilateral stimulation, stimulation of another cortical zone of or repeated stimulations sessions). The analysis of the different Val66Met profiles could also provide explanations regarding a lack of effect.

Although has been shown that tDCS can improve some motor activities in patients with chronic stroke [55–57], there is much variation between studies as well as across subjects [58, 59]. The present study should provide some answers regarding this variability by investigating the influence of the patients’ profiles and whether changes in cortical excitability are able to induce changes in patients’ gait. It has recently been shown that the combination of tDCS and robot-assisted gait training (RAGT) on a treadmill positively improved gait performance in patients with chronic stroke compared to the RAGT only [60], but not in patients with subacute stroke [61]. Andrade et al. (2017) showed that 10 sessions of tDCS can reduce the risk of falls in patients with chronic stroke [62]. Despite these positive results, many questions remain unanswered [63]. The results of the present study will constitute a useful base to determine the aspects of function that tDCS improves the most in patients with hemiparesis, and is an essential first phase towards the validation of this technique as a treatment in patients, coupled with task-oriented training (Additional file 1).

### Trial status

The trial is ongoing at the time of manuscript submission.

## Appendix

### Centre of mass (COM) displacement

Displacement of the COM during gait, the trajectory tracking and the balance tasks will be calculated using the following equation:

$$ CO{M}_x\kern0.5em =\kern0.5em \frac{m_1{x}_1+{m}_2{x}_2+\cdots {m}_i{x}_i}{M}\kern0.5em =\kern0.5em \frac{1}{M}{\sum}_{i=1}^N{m}_i{x}_i $$

where

*M* = whole body mass

*m*_*i*_ = mass of the ith segment = (whole body mass) × (mass fraction for ith segment from the anthropometrics data)

*x*_*i*_ = the x-coordinate of the centre of mass for the ith segment with respect to the calibration origin

*N* = the number of body segments

### Centre of pressure (COP) displacement

Displacement of the *COP*_*x*_ and *COP*_*y*_ during the balance tasks will be calculated using the following equation:

$$ CO{P}_x\kern0.5em =\kern0.5em \frac{M_y+{F}_x\ast {z}_{\mathrm{o}}}{F_Z} CO{P}_y\kern0.5em =\kern0.5em \frac{M_x+{F}_y\ast {z}_{\mathrm{o}}}{F_Z} $$

where

*F*_*x*_ = is the resultant of the forces on the x-axis

*F*_*y*_ = is the resultant of the forces on the y-axis

*F*_*z*_ = is the resultant of the forces on the z-axis

*M*_*x*_ = is the moment of force on the x-axis

*M*_*y*_ = is the moment of force on the y-axis

z_o_ = origin of the platform (the distance is given by the manufacturer)

## Additional file

Additional file 1: SPIRIT 2013 Checklist: recommended items to address in a clinical trial protocol and related documents. (DOC 119 kb)

## Abbreviations

3D: Three-dimensional
BAL: Instrumented balance task
CLI: Clinical evaluation
COM: Centre of mass
COP: Centre of pressure
EMG: Electromyography
FIM: Functional Independence Measure
GA: Gait analysis
MEP: Motor-evoked potential
RAGT: Robot-assisted gait training
SF36: The Short Form (36) Health Survey
tDCS: Transcranial direct current stimulation
TMS: Transcranial magnetic stimulation
